# Different endoscopic submucosal dissection techniques: a tailored approach

**DOI:** 10.1016/j.vgie.2024.12.007

**Published:** 2024-12-24

**Authors:** Shaimaa Elkholy, Abeer Awad, Hany Haggag, Karim Essam, Kerolis Yousef, Mohamed El Sherbiny

**Affiliations:** Gastroenterology Division, Internal Medicine Department, Faculty of Medicine, Cairo University, Cairo, Egypt

## Abstract

**Background and Aims:**

Endoscopic submucosal dissection (ESD) is an advanced, minimally invasive technique for the removal of GI lesions, offering several advantages over traditional methods, such as EMR. Although ESD facilitates en bloc resection and allows for precise pathologic assessment, its technical complexity underscores the need for tailored approaches. This video aims to provide a detailed explanation of the technical aspects of various ESD methods and to demonstrate the applicability of each technique in different clinical scenarios.

**Methods:**

This video provides a comprehensive, step-by-step technical review of classic ESD techniques as well as various ESD modifications. The most common modifications include pocket, modified pocket, bridge, multiple tunnels, hybrid, and traction-assisted ESD. Limitations of each method are discussed, and how to overcome these limitations using other methods is also addressed.

**Results:**

Pocket ESD is the most commonly used modification, creating a tunnel beneath the lesion. Modified pocket ESD follows a similar approach but combines the steps of dissection and making the circular incision simultaneously. Both methods are considered classic for esophageal lesions, although they also can be applied in colorectal ESD. Bridge ESD involves starting from the cecal side of the lesion, followed by the oral or anal side, and then connecting both sides. This approach provides effective countertraction and is particularly useful in rectal ESD, though it remains applicable in other locations. For circumferential lesions, the multiple tunnel technique is the optimal option for management. Hybrid ESD combines EMR and ESD techniques. Traction-assisted ESD, using simple tools such as a clip in line or band, can be applied in conjunction with all of the aforementioned methods.

**Conclusions:**

The adoption of a tailored ESD approach is crucial for improving procedural success rates, enhancing patient outcomes, and expanding the applicability of ESD to a broader range of complex lesions.

Endoscopic submucosal dissection (ESD) is a sophisticated technique designed for the minimally invasive removal of GI lesions, providing significant benefits over traditional methods like EMR.^1^ ESD allows for en bloc resection and precise pathologic evaluation, but its technical complexity and potential for adverse events highlight the need for customized approaches.^2^

As our understanding of these procedures advances, the future of submucosal endoscopy looks promising for both diagnostic and therapeutic applications.^3^ With the continued development of technologies such as endoscopic robotics, ESD may become the global treatment of choice for early GI neoplasms.^4^

This video presentation focuses on the technical aspects of ESD and discusses strategies for developing a tailored approach (Fig. 1A and B). ESD is a knife-based resection technique aimed at removing lesions en bloc, enabling thorough assessment of the margins and ensuring complete therapeutic resection (Fig. 2).

**Figure 1:**
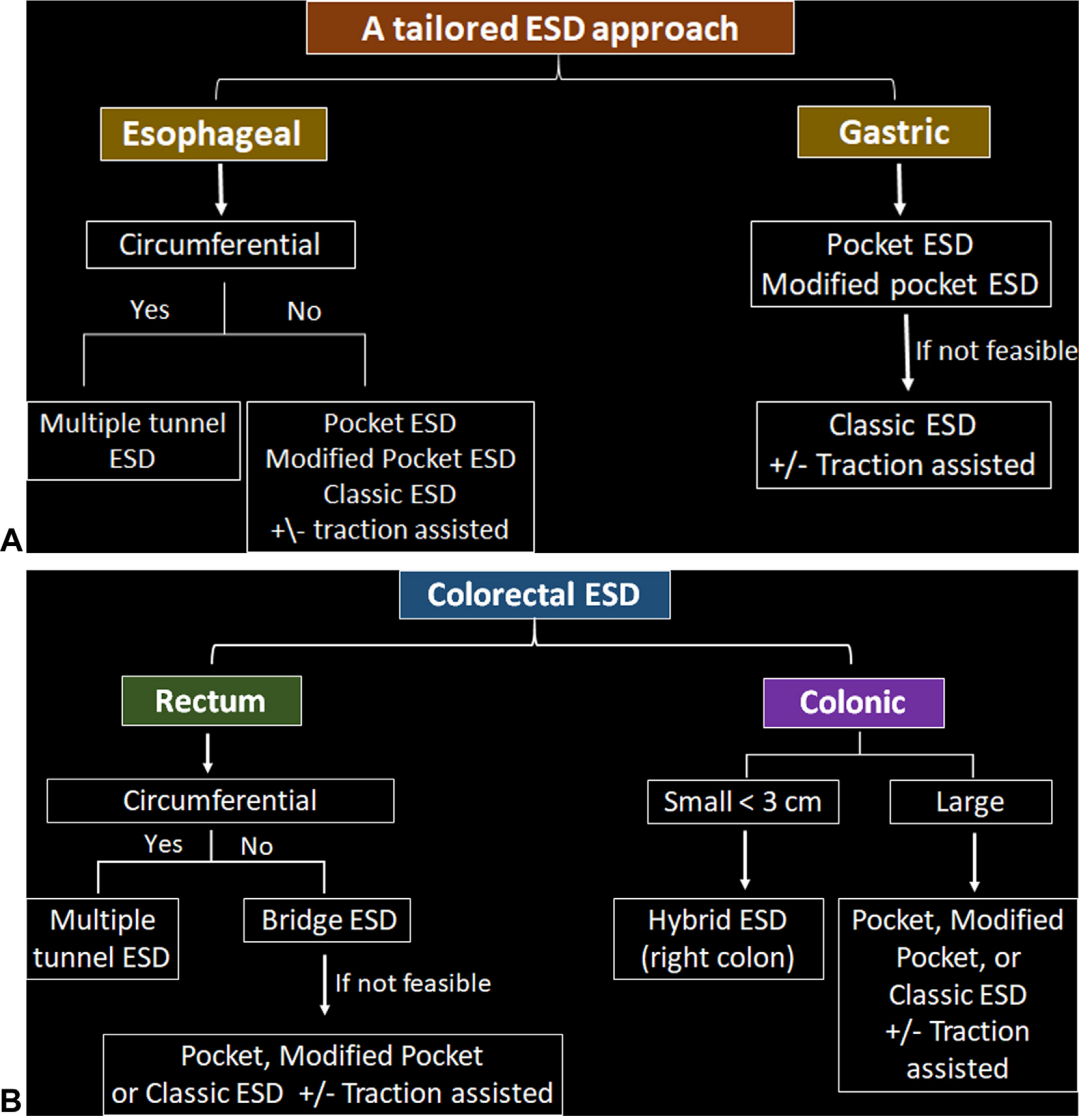
**A,** Tailored ESD approach for esophageal and gastric lesions: For circumferential esophageal lesions, multiple tunnel method can be used. For noncircumferential lesions, the pocket or modified pocket, or even classic ESD with or without traction could be used. For gastric lesions, the pocket or modified pocket technique is recommended. If not feasible, the classic ESD method with or without traction could be used. **B,** Tailored ESD approach for colorectal lesions: For circumferential rectal lesions, multiple tunnel method could be used. For noncircumferential lesions, bridge ESD is preferred; if not feasible, we can use either pocket or modified pocket or even classic ESD with or without traction. For colonic lesion <3 cm, hybrid ESD can be used if en bloc resection can be assured. In larger lesions, we can use pocket, modified pocket, bridge, or even classic ESD with or without traction. *ESD*, Endoscopic submucosal dissection.

**Figure 2:**
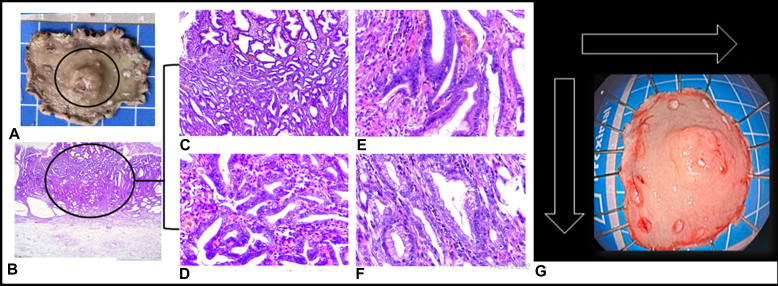
The principle of ESD is achieving en bloc resection, allowing for precise margin assessment and ensuring therapeutic resection. **A,** Gross specimen showing a well-demarcated, elevated lesion about 1 × 1 cm with a central depression. **B,** Histopathological section (H&E, ×40) highlighting the evaluation site with a free deep margin. **C and D,** Intramucosal carcinoma with fused glands, high-grade nuclear atypia, and mitotic activity (H&E, ×100 and ×200, respectively). **E and F,** Foveolar-type adenoma with high-grade dysplasia (H&E, ×400). **G,** Endoscopic view postresection, showing lesion orientation with resection margins (black arrows). *ESD*, Endoscopic submucosal dissection.

The classic ESD procedure involves several key steps: identifying the lesion, marking its perimeter, injecting a diluted dye (such as methylene blue or indigo carmine) into the submucosal layer, making a complete circular incision around the lesion's markings, and performing submucosal dissection to separate the lesion. Finally, the lesion is removed in 1 piece, ensuring en bloc resection^5^^,^^6^ (Video 1, available online at www.videogie.org, Fig. 3).

**Figure 3:**
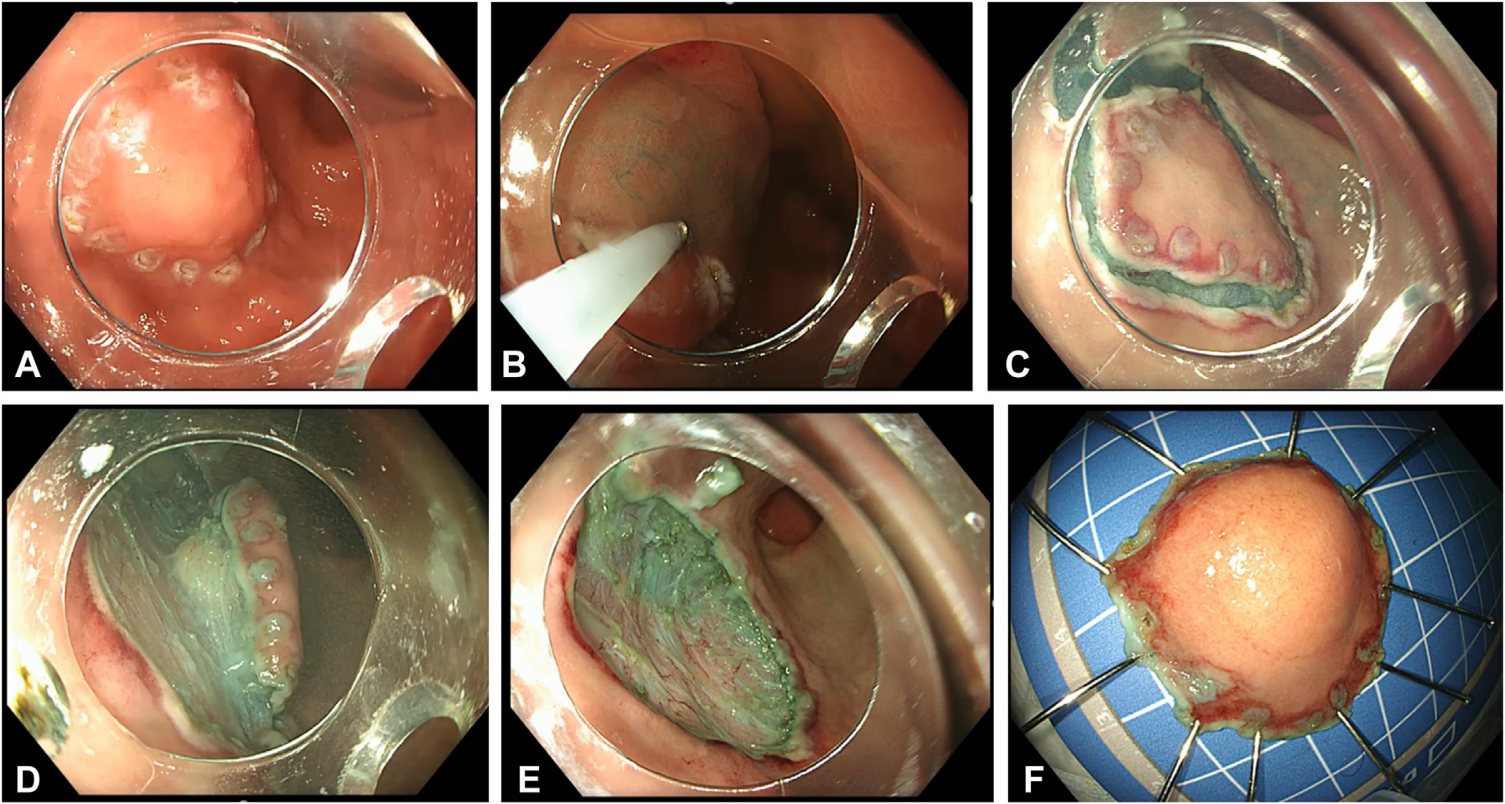
Steps of classic ESD: **A,** Marking all around the lesion using coagulation mode. **B,** Submucosal injection using a diluted dye as methylene blue or indigo carmine. **C,** Circular incision all around the lesion outside the marking. **D,** Submucosal dissection separating the lesion from the muscle layer. **E,** Hemostasis to the bed of the lesion. **F,** Retrieving the lesion en bloc resection. *ESD*, Endoscopic submucosal dissection.

There are several challenges encountered during ESD, such as lifting, stability, traction, difficult locations, adverse events, and the steep learning curve. As a result, the classic technique often is modified. To address these challenges and improve feasibility, various modified techniques have been developed, such as pocket ESD, modified pocket ESD, bridging ESD, multiple tunnel ESD, hybrid ESD, and traction-assisted ESD.^5^

### Pocket ESD

The concept of tunneling has significantly modified the classic ESD steps, making the procedure more feasible and technically versatile. One of the most well-known adaptations is pocket ESD, which involves creating a tunnel beneath the lesion, akin to placing a hand inside a pocket.^7^^,^^8^ The steps of pocket ESD are as follows: after marking the lesion and performing submucosal injection, a semilunar incision is made at either the oral or anal side of the lesion. Submucosal dissection is then carried out beneath the entire lesion. Once the dissection is complete, the circular incision is finalized from the outside, allowing for en bloc removal of the lesion (Video 1, Fig. 4)

**Figure 4:**
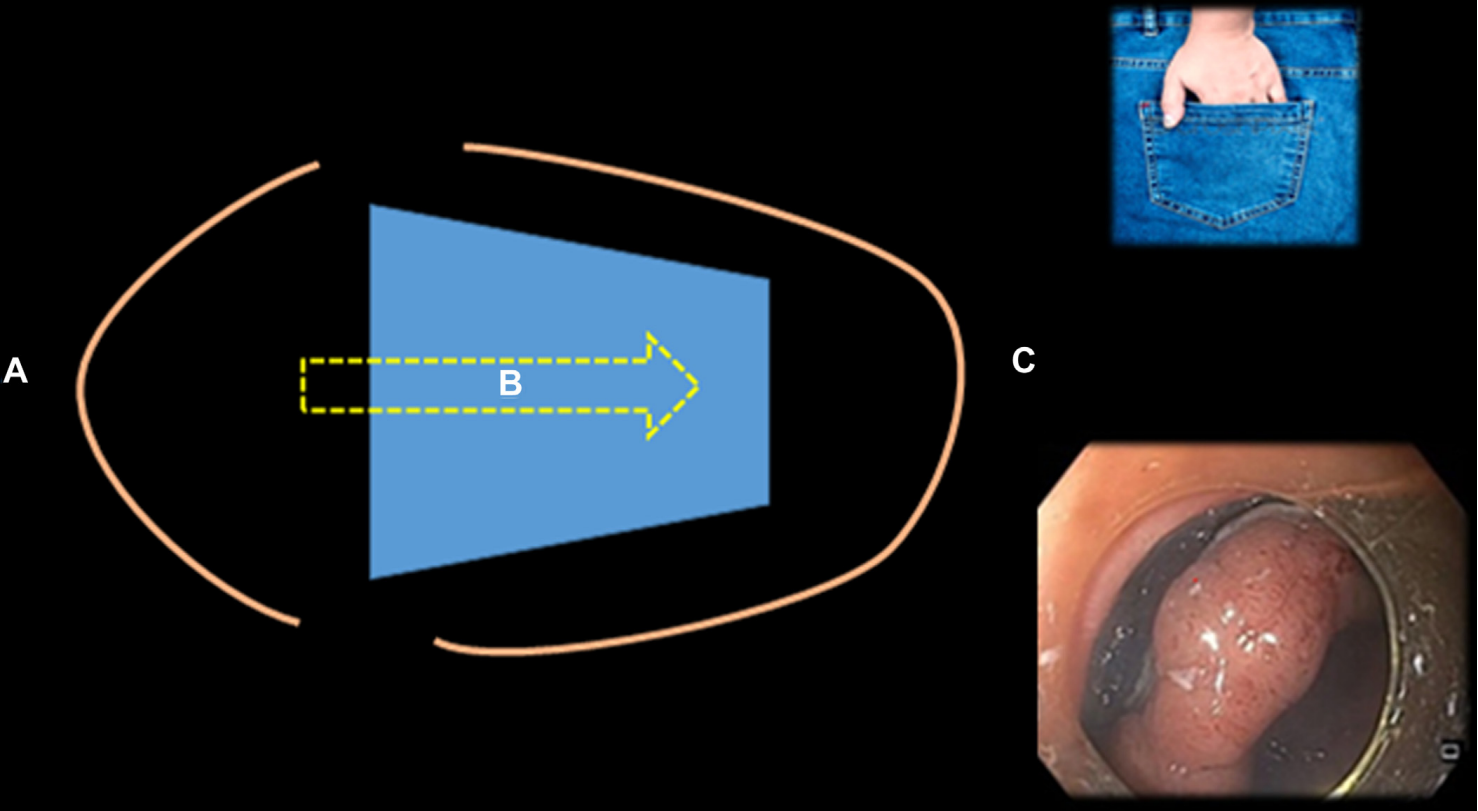
Steps of pocket ESD: **A,** Circular incision from the anal or oral side of the lesion. **B,** Creating a tunnel below the lesion by submucosal dissection. **C,** Completing the circular incision by cutting the lateral edges and cecal side of the lesion. *ESD*, Endoscopic submucosal dissection.

This technique offers numerous advantages, including improved scope stability, prevention of dye dispersion, and effective use of the cap to separate tissues and maintain alignment parallel to the muscle layer. In addition, it enhances visualization of blood vessels, enabling prophylactic coagulation, and is shown to have a great advantage in cases of submucosal fibrosis.^7^^,^^8^

Pocket ESD has demonstrated excellent outcomes in esophageal ESD and colorectal ESD, particularly when addressing laterally spreading tumors of the nongranular type.^7^^,^^9^ However, pocket ESD has some limitations. Its applicability in wide spaces, such as gastric lesions, can be challenging, although it remains feasible in certain cases. Another difficulty lies in determining when to stop dissection within the tunnel, not only at the end of the lesion but also along its lateral margins.

### Modified pocket ESD

The second modification, known as modified pocket ESD, is designed to address the limitations of the pocket ESD technique. Similar to pocket ESD, a tunnel is created just below the lesion. However, in modified pocket ESD, after entering the tunnel and initiating the dissection, the circular incision is periodically performed on both sides concurrently with the dissection.^10^ This approach opens the lesion like a book, allowing for the simultaneous completion of both the dissection and the circular incision (Video 1, Fig. 5)

**Figure 5:**
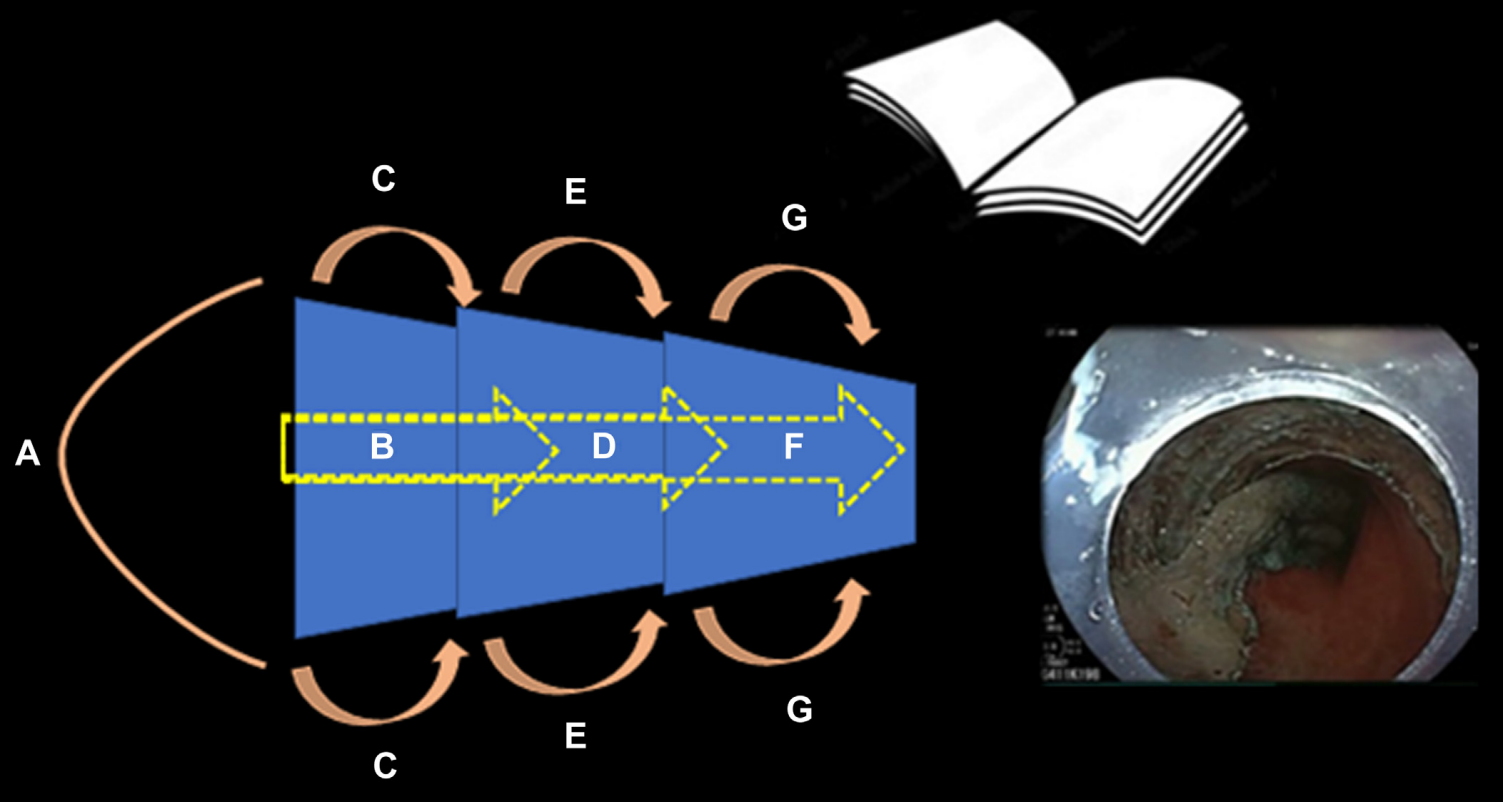
Steps of modified pocket ESD (open book): **A,** Circular incision from the anal or oral side of the lesion. **B,** Creating a tunnel below the lesion by submucosal dissection to the proximal part of the lesion. **C,** Performing the circular incision by cutting the lateral edges (on either side) of the proximal part of the lesion simultaneously with dissection. **D,** Going on with submucosal dissection to the middle part of the lesion. **E,** Performing the circular incision by cutting the lateral edges of the middle part of the lesion simultaneously with dissection. **F,** Performing the circular incision by cutting the lateral edges of the middle part. **G,** Completing the circular incision by cutting the lateral edges of the distal part of the lesion simultaneously with dissection. At the end, the lesion will hang down like an open book. *ESD*, Endoscopic submucosal dissection.

The modified pocket ESD technique is particularly helpful when maintaining the direction of dissection and identifying lesion margins is challenging. It is especially advantageous in colorectal ESD, particularly for large laterally spreading tumors.^10^ Furthermore, the hanging down of the lesion creates a spontaneous traction effect, facilitating further dissection.

A limitation of the modified pocket ESD technique is that the cecal end of the lesion often becomes obscured by the hanging lesion. However, this issue can typically be resolved by repositioning the patient.

### Bridge ESD

Bridge ESD is an innovative modification designed to address the limitations of both pocket and modified pocket ESD techniques. In this approach, the procedure begins on the cecal side of the lesion (the distal end), often during retroflexion of the scope. On the cecal side, either trimming the edge or creating a tunnel is performed. The procedure then continues on the anal or oral side of the lesion, where another tunnel is created. During dissection a hole becomes visible, representing the communication between both sides or tunnels, thereby forming a bridge.^11^ Once the bridge is created, the lesion falls under its own weight, providing effective traction. The procedure is completed by cutting the 2 pillars of the bridge (Video 1, Fig. 6).

**Figure 6:**
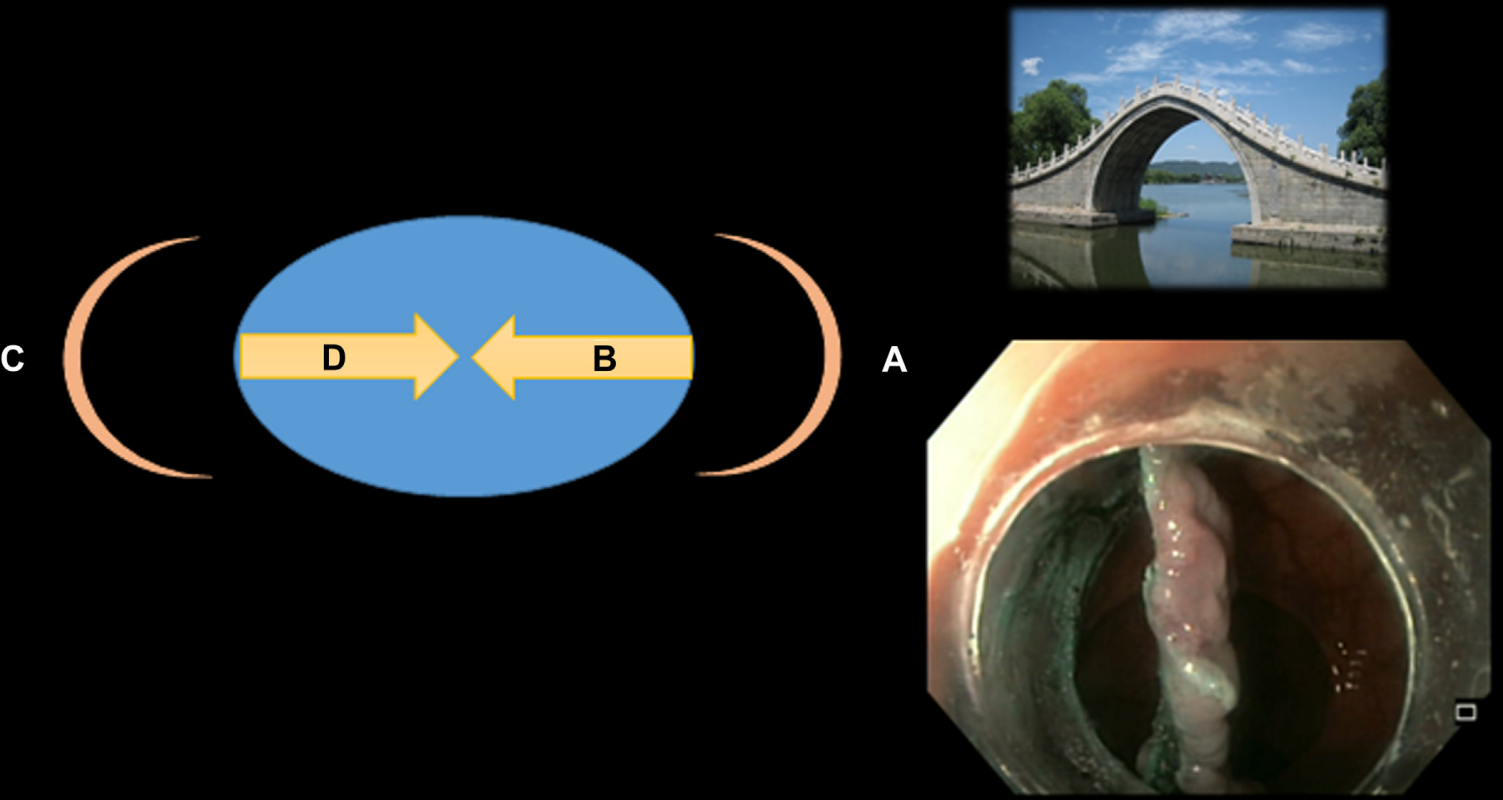
Steps of bridge ESD: **A,** Circular incision from the cecal side of the lesion on retroflexion. **B,** Submucosal dissection of the cecal side of the lesion, creating the first tunnel. **C,** Circular incision from the anal or oral side of the lesion. **D,** Submucosal dissection of the anal or oral side of the lesion, creating the second tunnel and connecting both tunnels together to form a bridge. *ESD*, Endoscopic submucosal dissection.

In addition, the scope can be passed through the bridge and retroflexed, which provides maximum countertraction and facilitates faster completion of the procedure. However, this maneuver should be performed with caution to avoid causing mechanical avulsion of the lesion^12^ (Video 1, Fig. 7).

**Figure 7:**
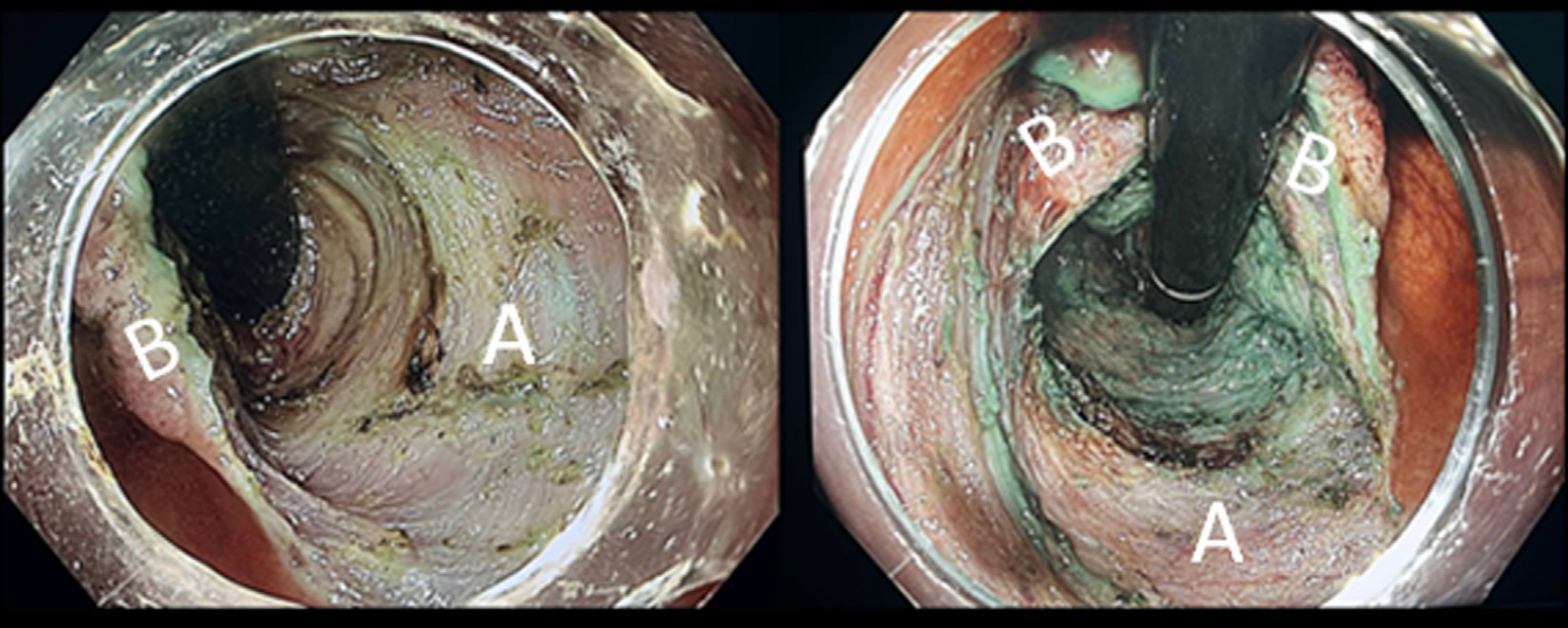
Endoscopic view during ESD. **A,** Lesion bed after resection. **B,** Dissected bridge providing countertraction. Retroflexion through the dissected bridge can enhance countertraction, improving dissection control. However, careful maneuvering is essential to avoid mechanical avulsion of the lesion, which may lead to adverse events such as bleeding or perforation. *ESD*, Endoscopic submucosal dissection.

Bridge ESD is particularly helpful for rectal or colonic lesions in which retroflexion of the scope is feasible. However, the technique has limitations when retroflexion cannot be achieved, such as in certain gastric, esophageal, or sigmoid ESDs. In such cases, the cecal side of the lesion can be directly dissected without retroflexion, but this should be approached cautiously because of the perpendicular angle to the muscle layer.

### Multiple tunnel ESD

Endoscopic management of circumferential or near-circumferential lesions is highly challenging and technically demanding. For such lesions, the previously mentioned techniques may not be sufficient. The multiple tunnel technique is considered a valuable approach for these cases.^13^ This technique involves creating multiple tunnels followed by connecting the tunnels together.^14^ The primary advantage of the multiple tunnel technique is that it prevents the lesion from obscuring the lumen by falling down, ensuring that the submucosal area remains clearly visualized throughout the procedure (Video 1, Fig. 8).

**Figure 8:**
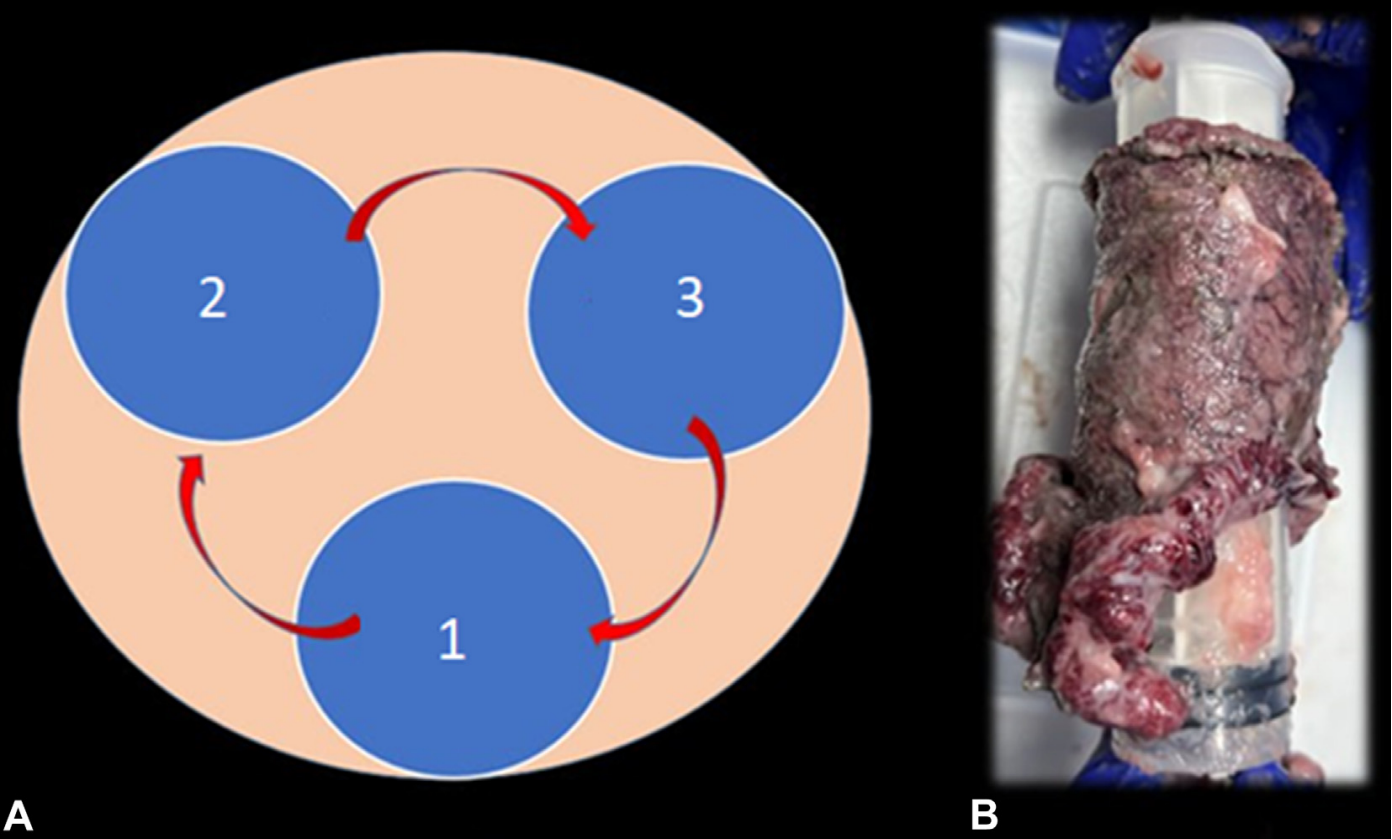
Multiple tunnel ESD for challenging circumferential lesions. **A,** Schematic representation of the multiple tunnel technique, where separate submucosal tunnels (1, 2, and 3) are created and subsequently connected to facilitate en bloc resection. **B,** Gross specimen of a circumferential lesion resected using the multiple tunnel approach. *ESD*, Endoscopic submucosal dissection.

### Hybrid ESD

The hybrid ESD technique combines the use of both a snare and a knife.^15^ The process begins with precise identification of the lesion margins, followed by a circular incision made around the lesion. Next, trimming of the circular incision is performed to shrink the lesion, enabling the snare to be anchored securely around it. This allows for en bloc resection to be achieved.

The principle of hybrid ESD is focused on achieving en bloc resection, which does not necessarily require classic ESD. As long as en bloc resection is possible, techniques such as EMR, ESD, or even hybrid method can be used. Hybrid ESD is particularly effective for lesions approximately 3 cm or smaller, especially those located on the right side of the colon.^16^ It offers a comparable safety profile with conventional ESD while significantly reducing procedure time (Video 1).

### Traction-assisted ESD

One of the most challenging steps in ESD is getting beneath the lesion, and traction can be very helpful. Obtaining traction during ESD is crucial, as it provides 2 significant benefits: it creates a clear visual field by flipping the mucosal flap and facilitates dissection by applying tension to the dissection plane. There are many commercially available traction devices, such as the multi-loop traction device (MLTD; Boston Scientific, Co, Ltd, Tokyo, Japan), Endo Trac (TOP, Tokyo, Japan), TRACMOTION retraction device (Fujifilm, Tokyo, Japan), and robotic device.^17^, ^18^, ^19^ However, many are expensive and not widely available. Simple traction methods, such as clip-in-line or band-assisted traction,^18^ can still be used with high efficacy, safety, and lower costs.

The clip-in-line method involves using a dental floss tied to a clip placed inside the working channel of the scope outside the patient. The clip is then attached to the edge of the lesion, and gentle traction is applied to the dental floss using an assisting hand. This method can be particularly useful in upper GI ESD, or when access to the exterior of the patient is easily achievable^20^ (Video 1).

Another technique involves using a band for traction, where one end of the band is clipped to the lesion's edge and the other to the contralateral bowel wall, known as band-assisted ESD. Band-assisted traction can provide multidirectional traction by using more than 1 band, and it can be applied without withdrawing the scope from the patient. This method is helpful in all ESD procedures, especially colonic ESD^21^ (Video 1).

The procedures demonstrated in Video 1 were done using an Olympus scope (GIF-1TH190, X11,500; Olympus, Tokyo, Japan), Fujifilm (760 CT), and Pentax (i10, Tokyo, Japan). Hyrbid knifes from Erbe (Tübingen, Germany) were used. An electrosurgical device, the VIO 3 (Erbe), was used with electrosurgical settings (Endocut Q3, 3, 3 and preciseSECT 4.5). Diluted methylene blue was used as the injection material, and the distal attachment cap was from Olympus.

### ESD tailored approach

Our proposed tailored ESD approach is illustrated in Figure 1A and B. For esophageal circumferential lesions, the multiple tunnel technique can be used. For noncircumferential lesions, the pocket or modified pocket ESD method is preferred. However, the classic method, with or without traction, also can be used.

For gastric lesions, if a tunnel can be created, the pocket or modified pocket technique is recommended. If not, the classic ESD method, with or without traction, is appropriate.

For circumferential rectal lesions, the multiple tunnel method is the standard approach. For noncircumferential lesions, the bridge ESD technique is preferred if feasible; otherwise, the pocket or modified pocket approach can be used.

For colonic lesions <3 cm, hybrid ESD is a practical choice. If not feasible, then the pocket, modified pocket, bridge, or classic ESD method with or without traction can be used.

## Conclusions

In ESD, there are no rigid rules. Meticulous planning and a tailored approach are essential for managing each lesion.

## Disclosure

All authors disclosed no financial relationships.

## Supplementary data

Video 1 In this video, we are focusing on the different technical aspects of endoscopic submucosal dissection (ESD) and how to have a tailored approach. ESD is a knife-based resection and the target of ESD is to remove the lesion en bloc Allowing for the assessment of both vertical and horizontal margins to ensure therapeutic resection. The classic steps of ESD are as follows: after identifying the lesion, marking is performed circumferentially using the knife in coagulation mode. This is followed by Submucosal injection with a diluted dye, such as methylene blue or indigo carmine. A circular incision is then made around the lesion, followed by submucosal dissection to separate the lesion from the muscle layer. Hemostasis is achieved in the lesion bed, and finally, the lesion is retrieved as en bloc resection. There are many challenges facing ESD; therefore, modifications have been done to the classic ESD to make it more applicable. The most famous modification is pocket ESD in which we create a tunnel below the lesion. We start with anal or oral side of the lesion followed by submucosal dissection creating a tunnel, then completing the circular incision by cutting the lateral edges and cecal side of the lesion afterwards. Modified pocket ESD is the same as pocket ESD; however, cutting the lateral edges of the lesion is performed simultaneously with submucosal dissection. At the end the lesion looks like an open book facilitating its dissection. Bridge ESD is when we start with the cecal side of the lesion followed by anal or oral side of the lesion connecting both sides together forming a bridge. Moreover, we can do retroflexion through the bridge carefully to cause more countertraction to the lesion facilitating its dissection. Multiple-tunnel ESD is classic for challenging circumferential lesions, in which we create multiple tunnels and then connecting them together to enable resection. Traction is highly beneficial in ESD and there are many commercially available devices. Although some are expensive, simple methods can still be used effectively. Hybrid ESD is the combination of the snare and knife. After performing a circular incision around the lesion using the knife, we anchor the snare all around the lesion to enable en bloc resection. Tailored ESD approach for esophageal and gastric lesions: for circumferential esophageal lesions, multiple tunnel method can be used. For noncircumferential lesions, the Pocket or modified pocket or even classic ESD with or without traction could be used. For gastric lesions, the pocket or modified pocket technique is recommended. If not feasible, the classic ESD method with or without traction could be used. Approach for colorectal lesions; for circumferential rectal lesions, multiple tunnel method could be used. For noncircumferential lesions, bridge ESD is preferred if not feasible we can use either pocket or modified pocket or even classic ESD with or without traction. For colonic lesions <3 cm, hybrid ESD can be used if en bloc resection can be assured. In larger lesions, we can use pocket, modified pocket or even classic ESD with or without traction.
